# The thermal stress response of *Aedes aegypti* and *Aedes albopictus* when exposed to rapid temperature changes

**DOI:** 10.1186/s13071-025-06951-4

**Published:** 2025-07-26

**Authors:** Hunter O. Covey, Randall Wilson, Yaizeth Gurrola-Mares, Joseph R. McMillan, Corey L. Brelsfoard

**Affiliations:** Department of Biological Sciences, Texas Tech University, 2901 Main St., Lubbock, TX 79409 USA

**Keywords:** Population control, Arboviruses, Temperature, Public health, Thermal stress

## Abstract

Autocidal mosquito control approaches are based on rearing mosquitoes in consistent and stable laboratory conditions; however, when adult mosquitoes are released in field settings, they have to rapidly respond to changes in temperature and thermal stress. To examine the effect of thermal stress on mosquitoes, *Aedes aegypti* and *Aedes albopictus* larvae were reared at either 22 °C (R22) or 28 °C (R28) and emerged adults were subsequently exposed to temperatures of 22 °C (E22), 28 °C (E28), 32 °C (E32), or 38 °C (E38). After the mosquitoes were subjected to these altered temperature profiles, we examined for effects on adult survivorship, respiration rates, and heat shock protein (HSP) expression. Reduced adult survivorship was observed when the rearing temperature was different than the adult exposure temperature. Respiration rates as a measure of thermal stress varied with rearing conditions and exposure temperatures, with notable declines observed as exposure temperatures increased. HSP gene expression was generally upregulated in response to thermal stress, with specific patterns differing by species and rearing conditions. Wing length measurements revealed no significant developmental differences across rearing temperatures, except for *Ae. aegypti* females. These findings highlight the impact of temperature on mosquito physiology and the potential impacts of rearing and environmental temperatures on the effectiveness of autocidal approaches for mosquito and disease control.

**Graphical Abstract:**

**Figure:**
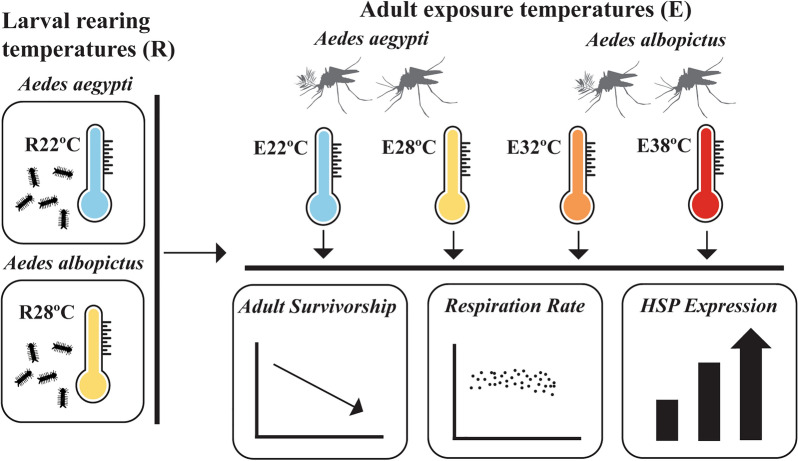

**Supplementary Information:**

The online version contains supplementary material available at 10.1186/s13071-025-06951-4.

## Background

Autocidal mosquito control approaches have gained a significant amount of attention as effective novel forms of species-specific population and disease control for container inhabiting *Aedes* species [1–4]. Autocidal approaches include the incompatible insect technique (IIT), sterile insect technique (SIT), *Wolbachia*-based population replacement, release of insects carrying a dominant lethal genes (RIDL), and autodissemination augmented by adult male mosquitoes (ADAM) [5–8]. All aforementioned approaches rely on releasing males, females, or a combination of both sexes into variable, natural environments that can survive, find mates, and in some cases produce offspring. The dynamic nature of field settings is at odds with the mosquito manufacturing processes in which food amounts, humidity, rearing space, and temperature are optimized and held constant during larval development [9–11]. Studies show that exposure to rapid temperature shifts can lead to thermal stress, such that released mosquitoes may experience reduced activity and decreased survival rates, and alter vector competence due to thermal stress [12–14].

The impact of thermal stress on ectothermic invertebrates has been evaluated in several ways including the examination life history traits (e.g., longevity, survivorship, fecundity, etc.), measuring metabolic rate, and the upregulation of heat shock protein genes (Hsps), which can aid in the acclimation to environmental stressors and protect from oxidative stress and cellular damage [14–19]. In this study, we examined the effects of larval rearing temperatures and the effects of exposing adult *Aedes aegypti* (Linus) and *Aedes albopictus* (Skuse) to a different temperature than the larval rearing temperatures. Experiments were conducted with *Ae. aegypti* and *Ae. albopictus* to examine how rearing temperature and exposing adults to different temperatures impacted survivorship and longevity, respiration rates, and heat shock protein gene expression as a measure of thermal stress.

## Methods

### Mosquito rearing

*Aedes albopictus* used in the described experiments consisted of the National Institutes of Health (NIH) National Institute of Allergy and Infectious Diseases (NIAID) strain from Gainesville, FL (MRA-804), contributed by Sandra A. Allan to BEI resources. *Aedes aegypti* used in these studies were from a colony strain collected from Corona, California. Eggs were hatched in 22 × 22 × 7.5 cm disposable plastic containers (Pactiv, Lake Forest, IL, USA) containing 500 mL aged liver powder solution (6 g liver powder/L) (Now Foods, Bloomingdale, IL). Larval pans and adult cages were housed in an incubator set to 28 ± 2.2 °C with a humidity of 85 ± 5% and a light (L):dark (D) cycle of 16:8 h. Eggs were hatched in a 1:1 solution of deionized water (DI) and fermented liver powder (0.6 g/L) (Now Foods, Bloomingdale, IL). After a 2-h egg hatch interval, first-instar larvae were separated into 21 cm × 21 cm × 7.5 cm disposable plastic rearing pans containing 500 mL of DI water at approximately 200 larvae per pan (Pactive, Lake Forest, IL). Larvae were provided a 60 g/L liver powder slurry for food ad libitum. Pupae were removed from larval rearing pans using a disposable plastic pipette and placed into 50 mL of DI water in a 140 mL plastic cups (Pactiv, Lake Forest, IL, USA). The plastic cup was then placed into a 24.5 × 24.5 × 24.5 cm BugDorm cage (MegaView Science Co., Taichung, Taiwan) where the pupae emerged into adults. Adults were provided with 10% sucrose solution ad libitum.

### Mosquito survivorship and longevity

To examine for an effect of thermal stress on adult survivorship and longevity, mosquitoes were reared at either 22 °C (R22) and 28 °C (R28) and were exposed to either 22 °C (E22), 28 °C (E38), 32 °C (E32), and 38 °C (E38) as adults. In brief, eggs were hatched using the same methods described in the mosquito rearing section. After a 2-h egg hatch interval, first-instar larvae were separated into 21 cm × 21 cm × 7.5 cm disposable plastic rearing pans containing 500 mL of DI water at approximately 200 larvae per pan (Pactive, Lake Forest, IL). Larvae were provided a 60 g/L liver powder slurry for food ad libitum. Pupae were transferred in 50 mL of DI water in 140 mL plastic cups (Pactiv, Lake Forest, IL, USA) to 24.5 × 24.5 × 24.5 cm BugDorm cages (MegaView Science Co., Taichung, Taiwan).

In total, 75 approximately 48-h-old male and female mosquitoes were aspirated using a mechanical aspirator and anesthetized by placing the aspirator tip with mosquitoes in plastic bag with a chloroform soaked cotton ball for 15–20 s. The adults were then transferred to 1.8 L (11.4 height × 23.7 cm diameter) plastic buckets modified to serve as mosquito cages (Airlite Plastics Co., Omaha NE). The buckets were modified into cages by adding a tubular stockenett (Richardson Products Inc., Frankfort, IL, USA) to provide access to the cage and a portion of the top of the lids removed and covered with no-see-um netting (Skeeta, Inc., Bradenton, FL, USA). Each cage type consisted of three to four replicates. The cages with mosquitoes were placed in an incubator set to the initial rearing temperature for 24 h. After the 24 h, the cages were moved to the respective exposure temperature. The cages were monitored daily for dead adults.

### Mosquito respiration as a measure of thermal stress

For respiration experiments, *Ae. aegypti* and *Ae. albopictus* were reared at either R22 or R28 as described in the previous section. Eggs were hatched using the same methods described in the mosquito rearing section. After a 2-h egg hatch interval, first-instar larvae were separated into 21 cm × 21 cm × 7.5 cm disposable plastic rearing pans containing 500 mL of DI water at approximately 200 larvae per pan (Pactive, Lake Forest, IL). Larvae were provided a 60 g/L liver powder slurry for food ad libitum. Pupae were transferred in 50 mL of DI water in 140 mL plastic cups (Pactiv, Lake Forest, IL, USA) to 24.5 × 24.5 × 24.5 cm BugDorm cages (MegaView Science Co., Taichung, Taiwan). Adults were provided with 10% sucrose solution ad libitum.

Mosquito respiration rates were determined by a Li-Cor gas analyzer (LI-6800–89) with an attached insect chamber (Chamber volume 49.9 cm^3^) (LICOR Corporate Offices—US, Lincoln, NE). Approximately 24 h post emergence, 50 males or females were aspirated from their respective cage and placed into the Li-Cor respiration chamber. The Li-Cor gas analyzer pump speed was set to auto, the flow rate was set to 400 μmol/s, the press valve was set to 0.0 kPa, H_2_O was set to on, the relative humidity was set to 85%, CO_2_ injector was set to on, the soda lime was set to scrub auto, and carbon dioxide concentration in the reference cell (CO_2__r) was set to a setpoint of 400 μmol^−1^. The adult mosquitoes were allowed to acclimate to the exposure temperature for 20 min before data collection began. A measurement of CO_2_ production was taken every 1 min for 120 min.

### Heat shock gene expression

Real-time quantitative polymerase chain reaction (qPCR) was performed to examine the expression of heat shock genes: *AeaHsp26*, *AeaHsp83*, and *AeaHsc70*. Primers for these genes are found in supplemental Supplementary Table S1 [20]. *Ae. aegypti* and *Ae. albopictus* female whole mosquitoes from each rearing and exposure temperature combination at 2 and 24 h postexposure were flash frozen using liquid nitrogen. RNA was extracted using Qiagen RNeasy kit following the manufacturer’s instructions. Following the RNA extraction, complementary DNA (cDNA) was made using a Lunascript cDNA synthesis kit (New England Biolabs, Ipswich, MA) following the manufacturer’s instructions. All amplifications were performed as two technical replicates using Platinum 10X SYBR Green qPCR SuperMix-UDG (ThermoFisher) on a Bio-Rad CFX96 qPCR system (Bio-Rad, Hercules, CA USA). Thermocycler amplification cycling conditions consisted of an initial denaturing step of 95 °C for 10 min followed by 40 cycles of 95 °C for 15 s, 54–60 °C for 1 min, and 72 °C for 30 s, and an elongation step at 72 °C for 8 min.

### Wing collection and measurement

*Ae. albopictus* and *Ae. aegypti* were reared at R22 and R28. Eggs were hatched using the same methods described in the mosquito rearing section. After a 2-h egg hatch interval, approximately 200 first-instar larvae of each species were placed into a separate 21 cm × 21 cm × 7.5 cm disposable plastic rearing pan containing 500 mL of DI water (Pactive, Lake Forest, IL). Larvae were provided a 60 g/L liver powder slurry for food ad libitum. Pupae were transferred in 50 mL of DI water in 140 mL plastic cups (Pactiv, Lake Forest, IL, USA) to 24.5 × 24.5 × 24.5 cm BugDorm cages (MegaView Science Co., Taichung, Taiwan). Once the mosquitoes reached adulthood, approximately 10–15 mosquitoes from both sexes (one for each sex and each rearing temperature) were aspirated and placed into a 2.5 mL Eppendorf tube containing mineral oil to prevent desiccation and stored at −20 °C. Wings were removed, placed on a slide, and imaged using an Leica S9 stereomicroscope and attached camera (Leica microsystems, Wetzlar, Germany). Wings were then measured from the base of the wing to the axial vein using the images and ImageJ software (vs. 1.54 m).

### Statistical analyses

Survivorship curves and log-rank analyses were performed using JMP Pro 17 (JMP Statistical Discovery LLC, Cary, NC). Comparisons of CO_2_ production from respiration trials was performed in R using a generalized estimation equation (GEE) model. Kruskal–Wallis tests followed by pairwise Wilcoxon tests were used to determine differences in the HSP gene expression and wing lengths using JMP Pro 17 (JMP Statistical Discovery LLC, Cary, NC).

## Results

### The effects of thermal stress on adult survivorship and longevity

**Fig. 1:**
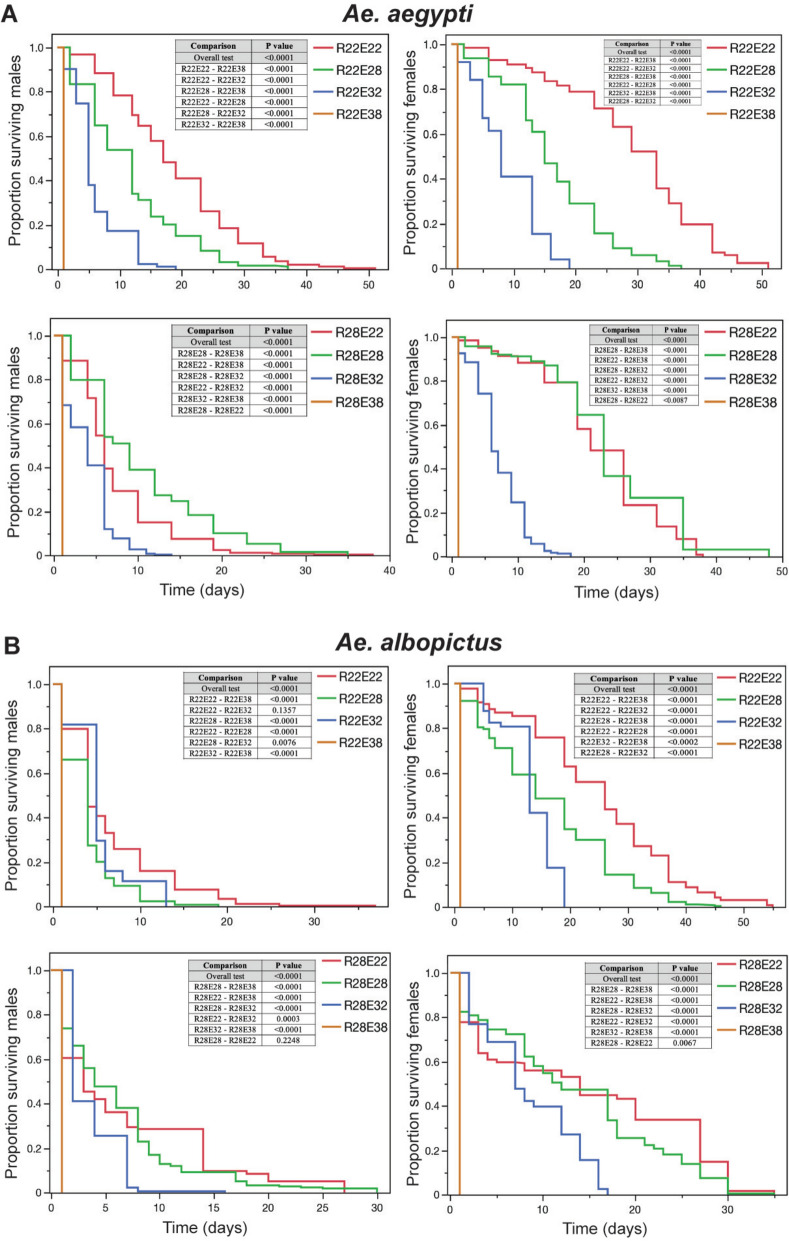
*Ae. aegypti* and *Ae. albopictus* mean survivorship after rearing at 22 °C and 28 °C (R22 and R28, respectively) and subsequent adult exposure at 22 °C, 28 °C, 32 °C, and 38 °C (E22, E28, E32, and E38, respectively). Distinct temperature treatments are represented by separate curves, indicating differential survival responses under thermal stress. Differences according log-rank survivorship tests between each treatment are shown in each plot

To examine for an effect of thermal stress on adult survivorship at different exposure temperatures, *Ae. aegypti* females and males were reared at 22 °C and 28 °C and exposed as adults to the temperatures 22 °C, 28 °C, 32 °C, and 38 °C. The survivorship of both *Ae. aegypti* and *Ae. albopictus* males and females was generally reduced when the rearing temperature was different than the exposure temperature (Bonferroni corrected log-rank tests, *P* < 0.008) (Fig. 1; Supplementary Table S2). In particular, as the difference in rearing and exposure temperature increased the longevity of *Ae. aegypti* and *Ae. albopictus* adults was reduced (Fig. 1). It is interesting to note that, at E38, *Ae. aegypti* and *Ae. albopictus* females and males did not survive longer than 24 h postexposure to 38 °C (Fig. 1). To determine whether the survivorship analysis was influenced by high early mortality rate in the 38 °C exposure treatments, the overall log-rank tests were repeated within groups without the 38 °C data and results were similar (Bonferroni corrected log-rank tests, *P* < 0.001).

### The effects of thermal stress on mosquito respiration

**Fig. 2:**
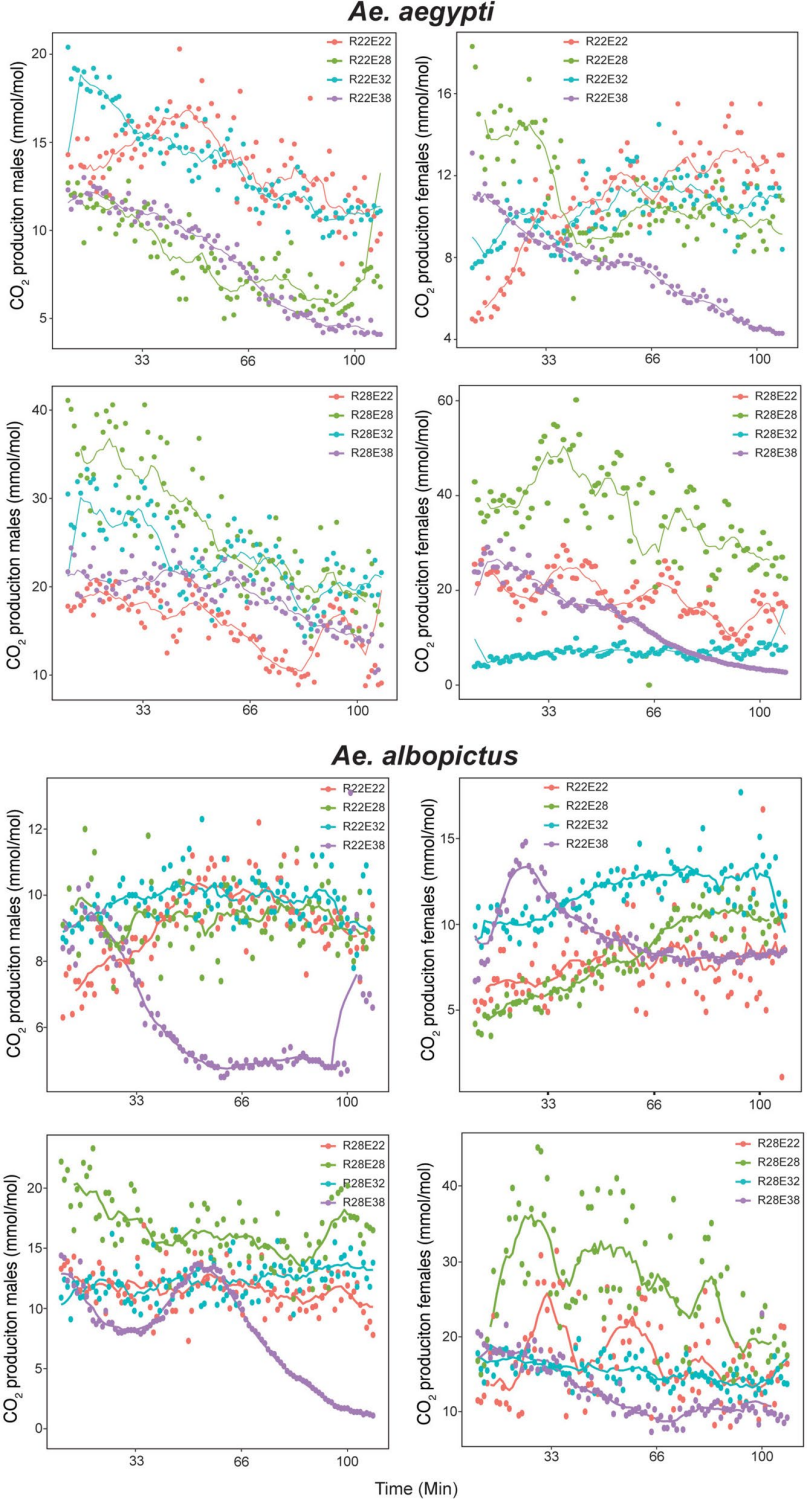
Adult respiration rates of *Ae. aegypti* and *Ae. albopictus* after rearing at 22 °C and 28 °C (R22 and R28, respectively) and subsequent adult exposure at 22 °C, 28 °C, 32 °C, and 38 °C (E22, E28, E32, and E38, respectively). CO_2_ (mmol/mol) production was measured for each rearing and exposure temperature combination for male and females over a 120 min time period

To examine the effect of thermal stress on mosquito respiration, *Ae. aegypti* and *Ae. albopictus* males and females were exposed to a different temperature (E22, E28, E32, and E38) than they were reared at (R22 and R28), and CO_2_ production was measured. We found a measurable reduction in respiration rates was observed when *Ae. aegypti* and *Ae. albopictus* males and females were exposed to rapid temperature changes (GEE, *P* < 0.0001) (Table 1). These results suggest the mosquitoes are experiencing thermal and ultimately metabolic stress associated with exposure to rapid temperature changes (Table 1 and Fig. 2). In both mosquito species, the greatest observed reduction in respiration rates was typically associated with a higher range of difference between the rearing and adult exposure temperature (e.g. E22 versus E38) (Fig. 2). To determine whether the observed differences in respiration rate were influenced by potential adult mortality in the 38 °C exposure treatments, the overall GEE comparisons were repeated within groups without the 38 °C data, and results were similar, suggesting that any potential adult mortality at this temperature did not bias the overall GEE comparisons (GEE, *P* < 0.001).

**Table 1:** GEE comparisons of respiration data for *Ae. aegypti* and *Ae. albopictus* females and males

Species	Rearing temperature	Exposure temperature	GEE			
			Estimate	SE	Wald	*P*-value
*Ae. aegypti*—males	R: 22 °C	Intercept	13.369	0.00198	45,520,992	< 0.001
		E: 28 °C	−4.74023	0.0026	3,322,525	< 0.001
		E: 32 °C	0.95896	0.00291	108,327	< 0.001
		E: 38 °C	−5.10075	0.00198	6,608,649	< 0.001
	R: 28 °C	Intercept	26.9	1.34 × 10^−5^	4.06 × 10^12^	< 0.001
		E: 22 °C	−11.8	2.05 × 10^−5^	3.30 × 10^11^	< 0.001
		E: 32 °C	−3.06	2.39 × 10^−5^	1.64 × 10^10^	< 0.001
		E: 38 °C	−8.32	1.67 × 10^−5^	2.48 × 10^11^	< 0.001
*Ae. aegypti*—females	R: 22 °C	Intercept	10.4	2.45 × 10^−6^	1.80 × 10^13^	< 0.001
		E: 28 °C	1.38	5.52 × 10^−6^	6.27 × 10^10^	< 0.001
		E: 32 °C	−0.539	4.15 × 10^−6^	1.68 × 10^10^	< 0.001
		E: 38 °C	−2.67	3.01 × 10^−6^	7.87 × 10^11^	< 0.001
	R: 28 °C	Intercept	136	1.68 × 10^−5^	4.48 × 10^12^	< 0.001
		E: 22 °C	−16.3	1.97 × 10^−5^	6.88 × 10^11^	< 0.001
		E: 32 °C	−29.0	1.73 × 10^−5^	2.82 × 10^12^	< 0.001
		E: 38 °C	−22.1	1.69 × 10^−5^	1.72 × 10^12^	< 0.001
*Ae. albopictus*—males	R: 22 °C	Intercept	8.93	6.34 × 10^−6^	1.98 × 10^12^	< 0.001
		E: 28 °C	0.332	6.80 × 10^−6^	2.38 × 10^9^	< 0.001
		E: 32 °C	0.738	8.67 × 10^−6^	7.24 × 10^9^	< 0.001
		E: 38 °C	−2.45	9.42 × 10^−6^	6.75 × 10^10^	< 0.001
	R: 28 °C	Intercept	17.0	9.69 × 10^−6^	3.09 × 10^12^	< 0.001
		E: 22°C	−5.55	1.04 × 10^−5^	2.85 × 10^11^	< 0.001
		E: 32°C	−4.67	9.69 × 10^−6^	2.32 × 10^11^	< 0.001
		E: 38 °C	−9.09	9.71 × 10^−6^	8.75 × 10^11^	< 0.001
*Ae. albopictus*—females	R: 22 °C	Intercept	7.73	1.12 × 10^−6^	4.78 × 10^13^	< 0.001
		E: 28 °C	0.286	1.56 × 10^−6^	3.38 × 10^10^	< 0.001
		E: 32 °C	3.91	4.82 × 10^− 6^	6.60 × 10^11^	< 0.001
		E: 38 °C	1.37	6.23 × 10^−6^	4.81 × 10^10^	< 0.001
	R: 28 °C	Intercept	25.9	3.10 × 10^−5^	7.01 × 10^11^	< 0.001
		E: 22 °C	−9.19	3.25 × 10^−5^	7.99 × 10^10^	< 0.001
		E: 32 °C	−10.6	3.10 × 10^−5^	1.17 × 10^11^	< 0.001
		E: 38 °C	−13.1	3.18 × 10^−5^	1.69 × 10^11^	< 0.001

E, exposed; GEE, generalized estimation equation; R, rearing; SE, standard error

### Expression of heat shock genes as a result of thermal stress

**Fig. 3:**
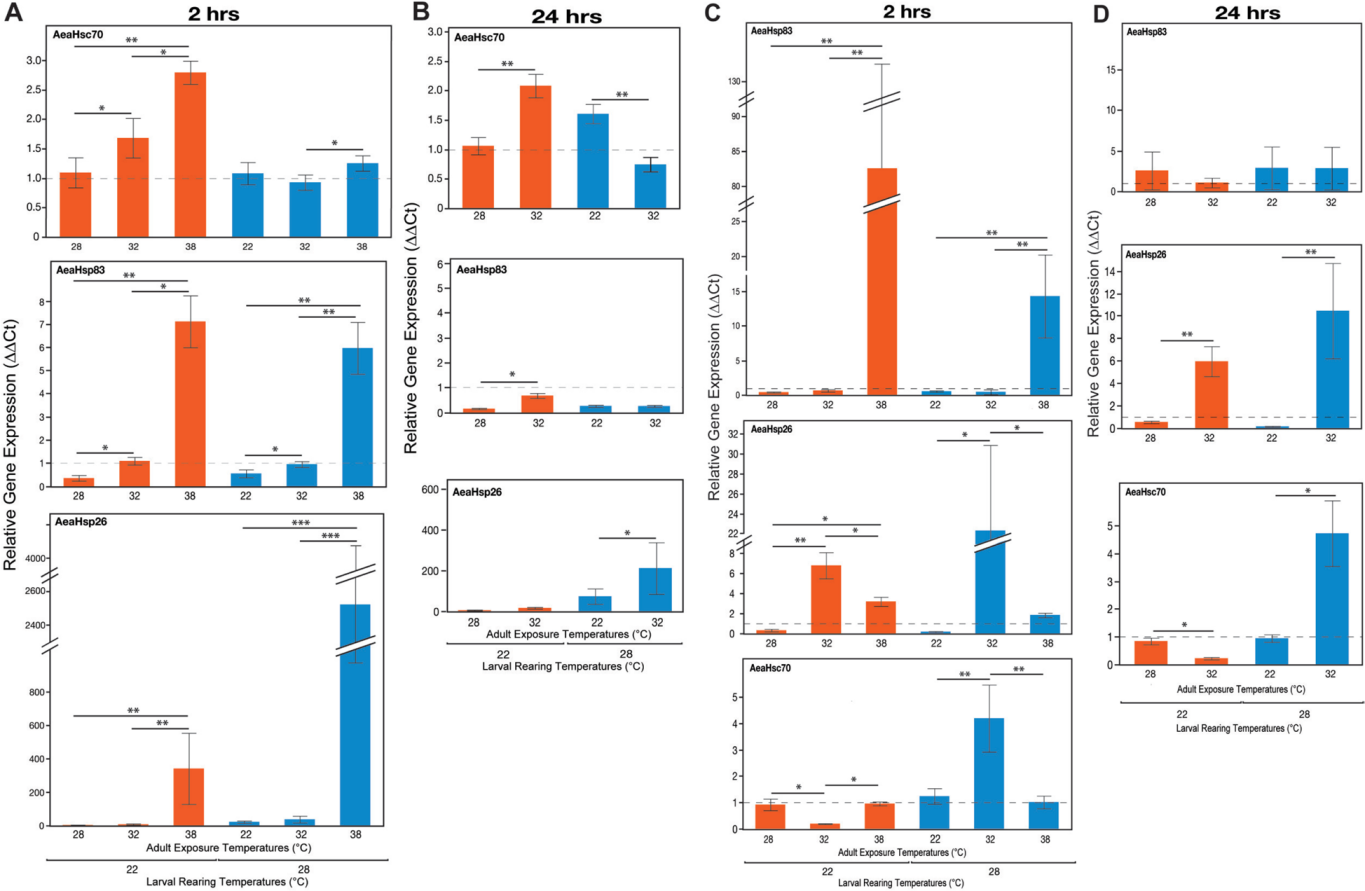
Mean HSP expression levels of *Aedes aegypti* females **A** 2 h and **B** 24 h and *Ae. albopictus* females **C** 2 h and **D** 24 h when reared at 22 °C and 28 °C (R22 and R28, respectively) and subsequent adult exposure at 22 °C, 28 °C, 32 °C, and 38 °C (E22, E28, E32, and E38, respectively). Lines with asterisks above the bars significant differences according to Bonferroni corrected pair-wise comparisons within each rearing temperature group (^*^P ≤ 0.01, ^**^P ≤ 0.001, and ^***^P ≤ 0.0001)

To further quantify the effect of rapid temperature changes and the resulting thermal stress, *Ae. aegypti* and *Ae. albopictus* adult females were exposed to a different temperature (E22, E28, E32, and E38) than their rearing (R22 and R28) temperature, and HSP gene expression was measured. We found that, when females experienced a rapid temperature change as adults, particularly at 38 °C, a collective increase in the expression of *AeaHsp83*, *AeaHsc70*, and *AeaHsp26* was observed (Kruskal–Wallis, *P* < 0.05) (Fig. 3). Furthermore, the changes in HSP expression levels were more pronounced in both species at 2 h postexposure rather than 24 h postexposure (Kruskal–Wallis, *P* < 0.05), suggesting that rapid temperature changes result in thermal stress (Fig. 3). All Kruskall–Wallis tests to examine for differences between each rearing temperature and post adult exposure times are shown in Supplementary Table S3.

### Effect rearing temperature on adult size

**Fig. 4:**
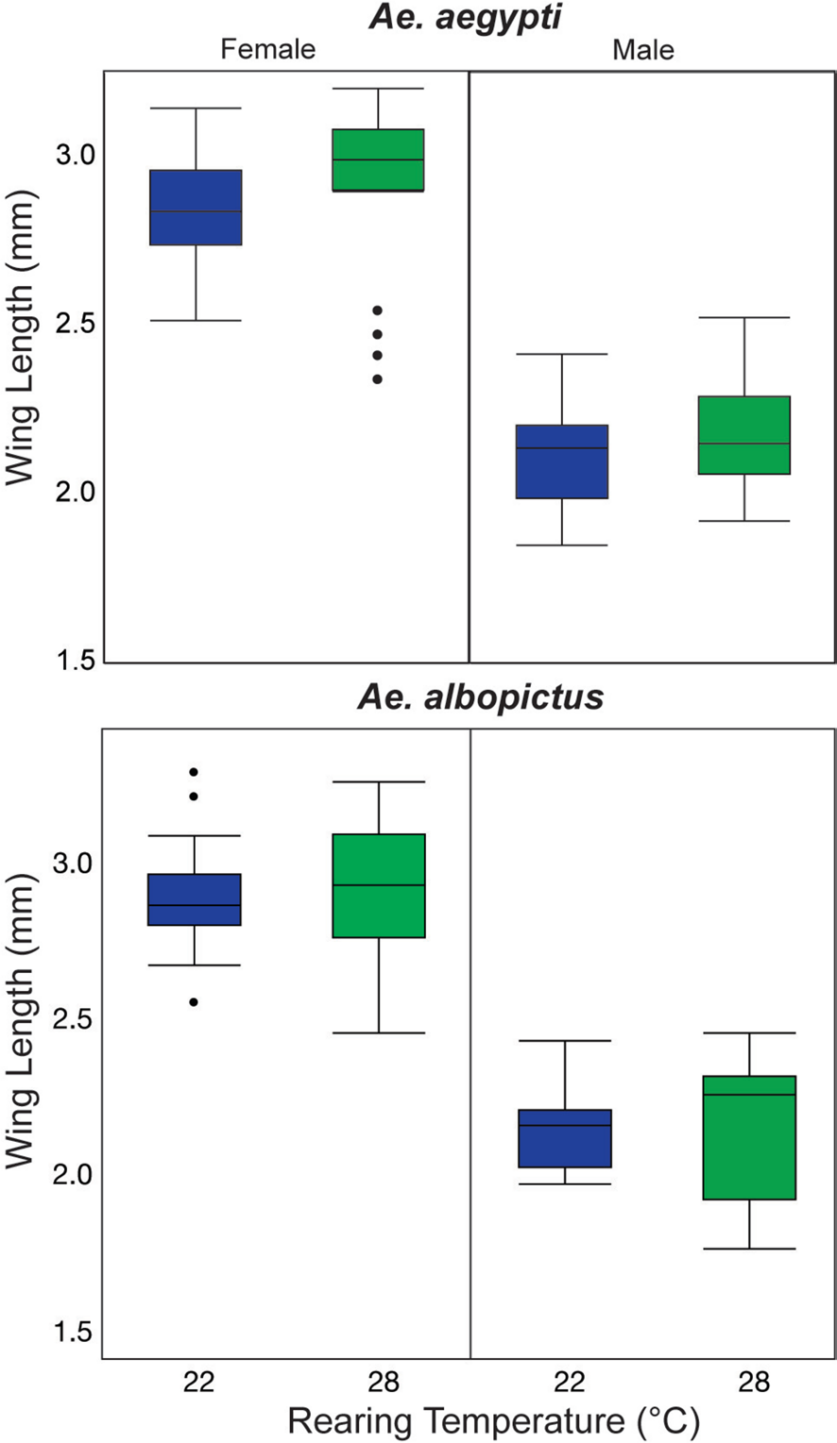
Wing length measurements for *Ae. aegypti* and *Ae. albopictus* males and females reared at 22 °C or 28 °C (R22 and R28). The upper and lower quartile values are the top and bottom of each box, and the median is represented by the line in each box. Error bars are standard deviation

When comparing the two different rearing temperatures, there was no observed impact on the wing length of *Ae. aegypti* (Kruskal–Wallis, chi-squared = 0.77, degrees of freedom [df] = 1, *P* = 0.38) or *Ae. albopictus* (Kruskal–Wallis, chi-square = 0.41, df = 1, *P* = 0.52) or within the sex of each species (Wilcoxon pair-wise tests, *P* < 0.05) (Fig. 4). These results suggest that there was no direct impact of rearing larvae at different temperatures resulting in adults of a different size, which could have impacted survivorship or respiration rates.

## Discussion

Our findings demonstrate that thermal stress significantly impacted the survivorship and longevity of *Ae. aegypti* and *Ae. albopictus* adults, with reduced survival observed when adults were exposed to a rapid temperature change. Both species exhibited optimal survivorship when rearing and exposure temperatures were the same (i.e., R22–E22 and R28–E28), suggesting a degree of thermal acclimation that enhances thermal tolerance. Conversely, exposure to temperatures that differed from rearing conditions, particularly at extreme temperatures such as 38 °C, dramatically reduced survival, with no individuals surviving beyond 24 h post-exposure. This aligns with previous studies indicating that exposure to high thermal extremes can exceed physiological limits, resulting in rapid mortality [14]. These results also suggest that thermal stress may be a potential indicator of survivorship rates when mosquitoes are exposed to temperature shifts in natural environments.

Interestingly, when using respiration as a proxy to measure thermal stress, we observed a unexpected reduction in respiration rates when mosquitoes reared at one temperature and later exposed to either lower or higher temperatures as adults. While an increase in temperature was hypothesized to correlate with higher respiration rates, *Ae. aegypti* and *Ae. albopictus* males and females exhibited a similar decrease in respiration rates when exposed to temperatures different from their rearing conditions [21]. The pattern of CO_2_ release in mosquitoes reflects their metabolic state, with discontinuous gas exchange (DGE) occurring during rest phases. When activity levels increase, DGE transitions to continuous gas exchange, reflecting heightened metabolic demands. This suggests that, under thermal stress, mosquitoes may adopt a strategy of reduced metabolic activity, potentially seeking out resting sites to conserve energy until conditions become more favorable. These findings align with previous studies that reported that mosquitoes prefer microhabitat resting areas that are cool and have higher humidity and where temperature variation is minimal before becoming active [22, 23]. Our respiration data further support the hypothesis that temperature shifts influence mosquito metabolism. When mosquitoes were maintained at the same temperature from larval to adult stages, respiration rates were higher compared with those experiencing a temperature shift. This suggests that any deviation from the rearing temperature imposes metabolic stress, leading to reduced activity and energy conservation [24, 25]. Exposure to higher temperatures in adulthood compared with larval conditions resulted in a dormancy-like response, lowering respiration rates and minimizing energy expenditure. While the E38 treatment had the most pronounced reduction in respiration rates, the observed dormancy-like response was not driven only by this temperature extreme but was also evident in each temperature shift from larval to adult exposure temperature.

As an additional measure of thermal stress, the expression of HSP was also examined. When comparing the expression of HSP relative to the treatment with the same rearing and adult exposure temperature there was an increase in HSP expression when there was a temperature shift higher than the rearing temperature. Consistently, the E38 treatment induced upregulation of heat shock genes across both species, indicating a thermal stress response associated with exposure to a detrimental temperature. The increase of expression of HSP associated with an increase in exposure temperature suggests that mosquitoes activate protective mechanisms to counteract thermal stress. Previous research has shown that *Ae. aegypti* and *Ae. albopictus* larvae exposed to high temperatures (e.g., 37 °C and 39 °C) developed increased thermotolerance upon re-exposure to even higher temperatures (43 °C and 45 °C) [18, 26]. This suggests that heat shock proteins (HSPs) play a crucial role in buffering the effects of thermal stress.

Additionally, we investigated whether larval rearing temperature influenced adult size by measuring wing length. No difference in wing length was observed when *Ae. aegypti* or *Ae. albopictus* were reared at 22 °C and 28 °C. This suggests that rearing temperatures of 22 °C and 28 °C had no impact on adult mosquito size, and that rearing at 22 °C or 28 °C ultimately did not result in any subsequent effects that could have biased the survivorship and respiration observations.

Here, we have demonstrated that rapid environmental temperature shifts result in thermal stress that can impact mosquito survivorship and metabolism. The resulting thermal stress response observed here may be similar what mosquitoes may experience when released as part of an autocidal approach. For example, if mosquitoes are reared at an consistent temperature of 28 °C in a insectary or factory and released into a natural environment where the temperature is 37–38 °C, the mosquitoes have to adapt quickly to this temperature shift. Ultimately, the resulting thermal stress from being released at this different temperature may impact the initial success of the releases depending upon the autocidal approach being utilized. These results suggest that the temperature at the time of mosquito release is a crucial factor to consider for autocidal approaches. Although ambient temperatures cannot be controlled, release timing can be optimized to improve dispersal and survivorship. To mitigate thermal stress, releases should ideally occur during the early morning or late evening in temperate and tropical regions where release temperatures are similar to their insectary or factory rearing temperatures. Furthermore, optimizing rearing and pre-release temperatures to match ambient conditions could enhance the fitness and effectiveness of released individuals. Evidence suggests that environmental conditions experienced at one life stage influence subsequent stages, emphasizing the importance of temperature adaptation across developmental phases [27]. Our experiments utilized laboratory-reared strains maintained for over 30 generations, raising the question of whether mosquitoes from natural populations with different genetic backgrounds might exhibit varying responses to temperature stress. Future studies should investigate the thermal adaptability of field-collected mosquitoes from diverse climatic regions to assess potential local adaptations. Moreover, the respiration experiments were unreplicated, and future studies could include additional replicates of strains with different genetic backgrounds.

Overall, our findings are similar to other previous studies and suggest that *Ae. aegypti* and *Ae. albopictus* have a narrow thermotolerance range and rely on genetic and behavioral responses to mitigate thermal stress [12, 16, 20, 26, 28, 29]. Understanding these adaptive strategies can improve the development and release of more robust mosquitoes for autocidal control methods.

## Supplementary Information

Additional file 1.

Additional file 2.

Additional file 3.

## Data Availability

Data supporting the main conclusions of this study are included in the manuscript.

## Abbreviations

R22: Reared at 22 °C
R28: Reared at 28 °C
E22: Exposed to 22 °C
E28: Exposed to 28 °C
E32: Exposed to 32 °C
E38: Exposed to 38 °C
DI: Deionized
HSP: Heat shock protein
SIT: Sterile insect technique
RIDL: Release of insects carrying a dominant lethal genes
ADAM: Autodissemination augmented by male mosquitoes
qPCR: Real-time quantitative polymerase chain reaction
DGE: Discontinuous gas exchange
