# Supplementary material for: The thermal stress response of Aedes aegypti and Aedes albopictus when exposed to rapid temperature changes
Source: Parasit Vectors. 2025 Jul 26;18:300. doi: 10.1186/s13071-025-06951-4 (PMC12297721; doi:10.1186/s13071-025-06951-4)
Supplement: Supplementary file 1 — Additional file 1. [file 13071_2025_6951_MOESM1_ESM.pdf]

Supplementary Table 1. Primer sequences for the HSG genes used for RT-PCR.

Gene	Direction	Sequence – 5' → 3'	Reference
Hsp26	Forward	5'-TTCAGCATCCTTCTCCTCGT-3'	Zhao et al. 2010
	Reverse	5'-CCACGAACTTCCAGGTCAAT-3'	
Hsp83	Forward	5'-AAGGCCGTTAAGGATCTGGT-3'	
	Reverse	5'-CGCTAGTGTGGGGAAGAGAG-3'	
Hsc70	Forward	5'-ATGAACCCAACCAACACCAT-3'	
	Reverse	5'-TGGAACTGATTTCCTCTGGG-3'	
AeaActin-F	Forward	5'- AGGACTCGTACGTCGGTGAC-3'	
	Reverse	5'- CGTTCAGTCAGGATCTTC-3'	
