# Supplementary material for: The thermal stress response of Aedes aegypti and Aedes albopictus when exposed to rapid temperature changes
Source: Parasit Vectors. 2025 Jul 26;18:300. doi: 10.1186/s13071-025-06951-4 (PMC12297721; doi:10.1186/s13071-025-06951-4)
Supplement: Supplementary file 2 — Additional file 2. [file 13071_2025_6951_MOESM2_ESM.pdf]

Supplementary Table 2. Log-Rank statistics comparing the survivorship of *Ae. aegypti* and *Ae. albopictus* when adults were exposed to different temperature profiles.

Species - sex	Rearing temperature	Log-Rank Test			
		Comparison	ChiSquare	DF	P value
Ae. aegypti - Males	R22°C	Overall test	1173	3	<0.0001
		R22E22 - R22E38	473	1	<0.0001
		R22E22 - R22E32	272.851	1	<0.0001
		R22E28 - R22E38	477	1	<0.0001
		R22E22 - R22E28	68.577	1	<0.0001
		R22E28 - R22E32	85.317	1	<0.0001
		R22E32 - R22E38	333.888	1	<0.0001
Ae. aegypti - Females	R22°C	Overall test	1024.8	3	<0.0001
		R22E22 - R22E38	479	1	<0.0001
		R22E22 - R22E32	345.504	1	<0.0001
		R22E28 - R22E38	480	1	<0.0001
		R22E22 - R22E28	199.344	1	<0.0001
		R22E32 - R22E38	346.615	1	<0.0001
		R22E28 - R22E32	119.379	1	<0.0001
Ae. aegypti - Males	R28°C	Overall test	733.3	3	<0.0001
		R28E28 - R28E38	491	1	<0.0001
		R28E22 - R28E38	374.106	1	<0.0001
		R28E28 - R28E32	180.348	1	<0.0001
		R28E22 - R28E32	73.267	1	<0.0001
		R28E32 - R28E38	241.916	1	<0.0001
		R28E28 - R28E22	31.252	1	<0.0001
Ae. aegypti - Females	R28°C	Overall test	1323.9	3	<0.0001
		R28E28 - R28E38	486	1	<0.0001
		R28E22 - R28E38	473.206	1	<0.0001
		R28E28 - R28E32	445.262	1	<0.0001
		R28E22 - R28E32	426.716	1	<0.0001
		R28E32 - R28E38	401.366	1	<0.0001
		R28E28 - R28E22	6.879	1	<0.0087
Ae. albopictus - Males	R22°C	Overall test	487.4	3	<0.0001
		R22E22 - R22E38	381.37	1	<0.0001
		R22E22 - R22E32	2.226	1	0.1357
		R22E28 - R22E38	284.853	1	<0.0001
		R22E22 - R22E28	42.105	1	<0.0001
		R22E28 - R22E32	7.12	1	0.0076
		R22E32 - R22E38	273.347	1	<0.0001
Ae. albopictus - Females	R22°C	Overall test	945.4	3	<0.0001
		R22E22 - R22E38	536.399	1	<0.0001
		R22E22 - R22E32	99.861	1	<0.0001
		R22E28 - R22E38	490.415	1	<0.0001
		R22E22 - R22E28	62.809	1	<0.0001
		R22E32 - R22E38	356	1	<0.0002
		R22E28 - R22E32	14.941	1	<0.0001
Ae. albopictus - Males	R28°C	Overall test	418.4	3	<0.0001
		R28E28 - R28E38	260.45	1	<0.0001
		R28E22 - R28E38	197.16	1	<0.0001
		R28E28 - R28E32	29.082	1	<0.0001
		R28E22 - R28E32	13.323	1	0.0003
		R28E32 - R28E38	404	1	<0.0001
		R28E28 - R28E22	1.473	1	0.2248
Ae. albopictus - Females	R28°C	Overall test	598.4	3	<0.0001
		R28E28 - R28E38	296.233	1	<0.0001
		R28E22 - R28E38	292.921	1	<0.0001
		R28E28 - R28E32	61.715	1	<0.0001
		R28E22 - R28E32	57.643	1	<0.0001
		R28E32 - R28E38	423	1	<0.0001
		R28E28 - R28E22	7.358	1	0.0067
