# Supplementary material for: The thermal stress response of Aedes aegypti and Aedes albopictus when exposed to rapid temperature changes
Source: Parasit Vectors. 2025 Jul 26;18:300. doi: 10.1186/s13071-025-06951-4 (PMC12297721; doi:10.1186/s13071-025-06951-4)
Supplement: Supplementary file 3 — Additional file 3. [file 13071_2025_6951_MOESM3_ESM.pdf]

Supplementary Table 4. Kruskall-Wallis tests examining for differences between HSG expression levels at each rearing temperature (R22 and R28).

Species	Rearing temperature	Time Point	Gene	Kruskal-Wallis Test		
				ChiSquare	DF	P
Ae. aegypti	R22°C	2hrs	AeaHSP26	7.46	2	0.0240*
			AeaHSP83	12.02	2	0.0025*
			AeaHSC70	9.62	2	0.0081*
		24hrs	AeaHSP26	3.1527	1	0.0758
			AeaHSP83	6.8182	1	0.0090*
			AeaHSC70	5.7709	1	0.0163*
	R28°C	2hrs	AeaHSP26	4.4857	2	0.1062
			AeaHSP83	10.82	2	0.0045*
			AeaHSC70	2.66	2	0.2645
		24hrs	AeaHSP26	0.8836	1	0.3472
			AeaHSP83	0.0109	1	0.9168
			AeaHSC70	6.8182	1	0.0090*
Ae. albopictus	R22°C	2hrs	AeaHSP26	11.58	2	0.0031*
			AeaHSP83	9.62	2	0.0081*
			AeaHSC70	9.38	2	0.0092*
		24hrs	AeaHSP26	6.8182	1	0.0090*
			AeaHSP83	0.8836	1	0.3472
			AeaHSC70	6.8182	1	0.0090*
	R28°C	2hrs	AeaHSP26	12.5	2	0.0019*
			AeaHSP83	5.78	2	0.0556
			AeaHSC70	7.44	2	0.0242*
		24hrs	AeaHSP26	6.8182	1	0.0090*
			AeaHSP83	0.0982	1	0.8345
			AeaHSC70	6.8182	1	0.0090*

* $P < 0.05$
